# Persistent paramyxovirus infections: in co-infections the parainfluenza virus type 5 persistent phenotype is dominant over the lytic phenotype

**DOI:** 10.1099/jgv.0.001916

**Published:** 2023-11-14

**Authors:** Richard E. Randall, Dan F. Young, David J. Hughes, Steve Goodbourn

**Affiliations:** ^1^​ School of Biology, Centre for Biomolecular Sciences, BMS Building, North Haugh, University of St Andrews, St Andrews, Fife, KY16 9ST, UK; ^2^​ Section for Pathogen Research, Institute for Infection and Immunity, St George’s, University of London, London SW17 0RE, UK

**Keywords:** paramyxovirus, persistence, PIV5

## Abstract

Parainfluenza virus type 5 (PIV5) can either have a persistent or a lytic phenotype in cultured cells. We have previously shown that the phenotype is determined by the phosphorylation status of the phosphoprotein (P). Single amino acid substitutions at critical residues, including a serine-to-phenylalanine substitution at position 157 on P, result in a switch between persistent and lytic phenotypes. Here, using PIV5 vectors expressing either mCherry or GFP with persistent or lytic phenotypes, we show that in co-infections the persistent phenotype is dominant. Thus, in contrast to the cell death observed with cells infected solely with the lytic variant, in co-infected cells persistence is immediately established and both lytic and persistent genotypes persist. Furthermore, 10–20 % of virus released from dually infected cells contains both genotypes, indicating that PIV5 particles can package more than one genome. Co-infected cells continue to maintain both genotypes/phenotypes during cell passage, as do individual colonies of cells derived from a culture of persistently infected cells. A refinement of our model on how the dynamics of virus selection may occur *in vivo* is presented.

## Introduction

Parainfluenza virus type 5 (PIV5; previously known as simian virus 5; species *Mammalian rubulavirus 5*) is a prototypic member of the subfamily *Rubulavirinae* in the family *Paramyxoviridae* that belongs to the order Mononegavirales. The family *Paramyxoviridae* is a large group of vertebrate viruses that are primarily known for their ability to cause acute infections in humans (e.g. mumps, measles and parainfluenza virus infections), mammals (e.g. canine distemper virus and rinderpest), poultry (e.g. Newcastle disease virus) and fish (e.g. pacific salmon paramyxovirus, reviewed in [1]). However, under certain conditions some paramyxoviruses can also establish persistent (or prolonged) infections [2] that, whilst they may only last for a matter of weeks or months, last longer than would be expected from a prototypical acute infection in immunocompetent individuals (usually 2–3 weeks) [3]. Often, prolonged/persistent infections are observed in immunocompromised patients [3–5], although immunocompetent patients may also become persistently infected [2]. For example, outbreaks of parainfluenza virus infections that occurred in healthy young adults after weeks of isolation at the South Pole were probably due to persistent low-level shedding by some individuals [6]. Occasionally, certain persistent paramyxovirus infections can last for years or even for the lifetime of the individual, which may lead to chronic and sometimes fatal disease. For example, sub-acute sclerosing panencephalitis (SSPE), which is invariably fatal, is caused by a persistent measles virus infection of the brain [7] and chronic kidney disease in cats is associated with feline paramyxovirus [8, 9]. In general, the ability of viruses to establish persistent infections can be of clear benefit to the virus, since persistently infected individuals may act as reservoirs for the virus, reseeding it back into the community over a prolonged period. Furthermore, if persistent infection significantly influences the epidemiology of a virus it would be expected that viral traits that facilitate the establishment of persistence would be selected during virus evolution [3].

PIV5 (for a general review on the biology and molecular biology of PIV5 see [10]) is clearly a very successful virus, in that, unusually for most viruses, it readily crosses species barriers. It has been isolated from, and/or nucleotide sequences have been detected in, humans [11–14], monkeys [15–17], dogs [18], cattle [19, 20], pigs [21–25], tigers [26, 27], lesser panda [27] and pangolins [28], and there is some evidence that PIV5 may infect cats, hamsters, rats and guinea pigs [15]; a very closely related virus, Alston virus, has also been isolated from an Australian pteropid bat colony [29]. However, despite the fact that PIV5 infections appear to be endemic in at least dogs, cattle, pigs and humans, its association with disease is not clear. For example, although neutralizing antibodies and T cell responses to PIV5 have been detected in a high percentage of human blood [13, 15, 30, 31], there is no clear association of PIV5 with human respiratory disease. Similarly, although PIV5 has been linked to acute respiratory and diarrhoea symptoms in piglets [24, 32, 33] and calves [34], it has yet to be proven that PIV5 is a significant cause of illness in these animals. The best correlation between PIV5 and acute illness is in dogs, in which PIV5 is one of the causes of kennel cough (the virus is often called canine parainfluenza virus in veterinary circles). However, multiple pathogens are usually associated with kennel cough, making it difficult to ascribe symptoms to PIV5 alone [18]. There is some suggestion that persistent PIV5 infections may be linked to chronic disease. For example, PIV5 has also been detected in the brains of cattle with neurological symptoms [20], but the link to disease remains unproven. Interestingly, PIV5 can cause unsuspected persistent infections of cultured cells [35, 36], and is likely to establish persistent infections *in vivo* [11, 37]. We speculate that such persistent infections may be important in maintaining PIV5 in human and animal populations.

PIV5 has a non-segmented negative-sense RNA genome of 15 246 bases, which has 7 genes (3′-N-P/V-M-F-SH-HN-L-5′) that encode 8 proteins (the P and V proteins are made from differentially transcribed forms of the same gene), and has non-coding leader (Le) and trailer (Tr) sequences at its 3′ and 5′ ends, respectively (reviewed in [10]). The viral RNA-dependent RNA polymerase (RdRp) complex recognizes the genomic Le promoter elements and drives transcription of viral mRNAs as a gradient [38] through recognition of *cis*-acting gene start (Gs) and gene end (Ge) elements that flank each gene. The RdRp also initiates replication of a full-length antigenome from Le, and then a full-length genome from Tr on the antigenome. Although the mechanisms that enable the RdRp to ignore the *cis-*acting elements of the transcription units are not fully understood, it is thought that as the intracellular concentration of NP increases so encapsidation of the newly synthesized viral RNA begins, and is the critical step in the switch from transcription to replication (for a general review of the molecular biology of paramyxoviruses, including PIV5, see [1, 10].

We have been studying PIV5 as a model for viral persistence [39]. Our recent research has shown that the properties of the RNA polymerase subunit P protein influence whether PIV5 establishes lytic or persistent infection. Infection of cultured cells with certain strains of PIV5 induces cell death (lytic), whilst infection with other PIV5 strains (including the reference laboratory strain, W3A) leads to the immediate establishment of virus persistence without the cells going into crisis and with the cells being able to be readily passaged. In individual persistently infected cells the virus fluxes between repressed and active states leading to the continuous long-term production of infectious virus. The switch between persistent and acute infection is regulated by the phosphorylation state of P. Phosphorylation of key serine residues [e.g. a serine (S) at position 157 of P] by cellular kinases is associated with negative regulation of PIV5-P activity that leads to the reduction of viral RNA and protein synthesis and the establishment of persistence. PIV5 strains that have a single amino acid substitution in the target phosphorylation sites (e.g. serine to phenylalanine at position 157) cause lytic infections in tissue culture cells. In mice, these lytic strains replicate to higher titres than the persistent ones and cause greater infiltration of immune cells into infected lungs but are cleared more rapidly [39].

Paramyxovirus virions are highly pleiomorphic, with typical spherical forms ranging from 50 nm to more than 500 nm in diameter [40]. Embedded in the envelope of the virion, which egresses by budding from the host cell membrane, are the attachment haemagglutinin–neuraminidase (HN) and fusion (F) glycoproteins. Also inserted into the lipid envelope of PIV5 and some other rubulaviruses are copies of the small hydrophobic (SH) protein. The matrix (M) protein, which underlies the lipid membrane, is required for the structural integrity of the virion, co-ordinating the surface proteins with the nucleocapsid. The nucleocapsid, which is remarkably stable, is a helical protein : RNA structure in the core of the particle in which the nucleocapsid protein (NP) encapsidates the negative single-stranded non-segmented genome. Copies of the virally encoded RNA-dependent RNA polymerase (RdRp) complex, containing the large (L) protein and phosphoprotein (P), are found associated with the nucleocapsid. In PIV5, and some other rubulaviruses, copies of the V protein, the viral interferon antagonist, are also associated with the nucleocapsid (for reviews see [1, 10, 40]).

The varying size and pleiomorphic nature of paramyxovirus virions can at least partially be explained by the observation that large numbers of virions contain multiple genomes. Using low-resolution cryo-electron tomography, Loney *et al*. reported that virions of Sendai virus, which can vary in size from 110 to 540 nm, can contain up to six genomes [41]. Furthermore, some of their data were best explained if defective interfering genomes were packaged alone or co-packaged with full-length genomes. Rager *et al*. showed that multiple genomes of measles virus could be co-packaged [42] and evidence presented by Goffet *et al*. showed that whilst the majority of NDV particles contained a single genome, up to 25 % may contain two or more genomes [43].

Here we have investigated whether in co-infections the persistent or lytic phenotype is dominant. Not only do we show that the persistent phenotype is dominant, but we also show that in persistently co-infected cells both genomes persist and that up to 17 % of virions released from co-infected cells contain both genotypes. The biological implications of these observations are discussed.

## Results

### Characterization of persistent and lytic reporter viruses

**Fig. 1.**
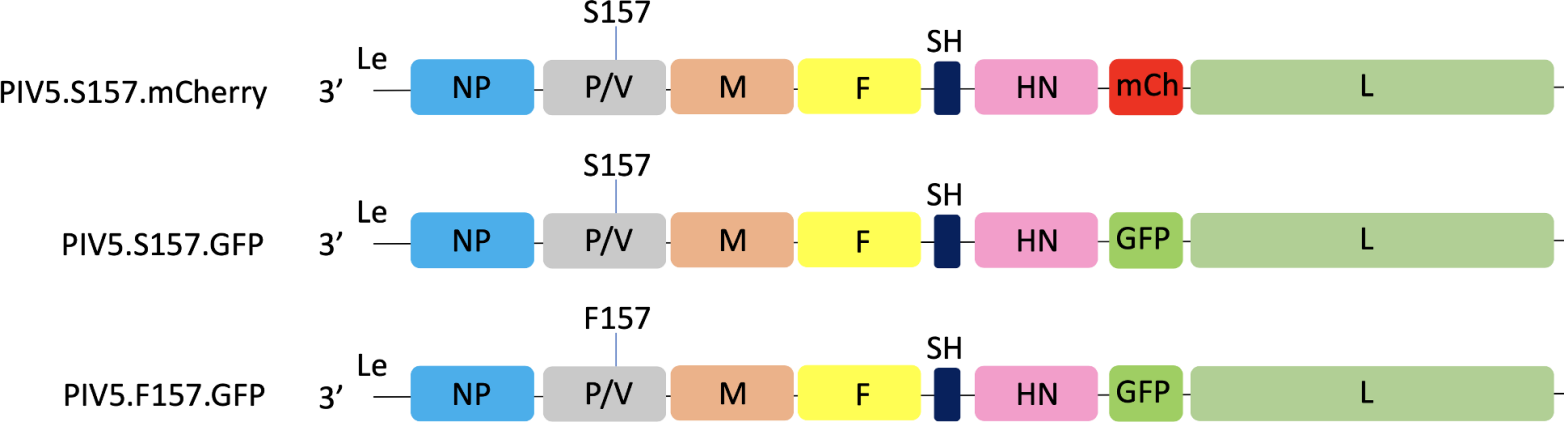
Reporter viruses used in this work. The construction of the mCherry and GFP viruses used in this work is described in the Methods section. The figure shows the three recombinant genomes, PIV5.S157.mCherry, PIV5.S157.GFP and PIV5.F157.GFP. Viral genes are indicated as NP (blue), P (grey), M (brown), F (yellow), SH (dark blue), HN (pink) and L (light green). Alternative versions of the P gene (either S157 or F157) are indicated above the P gene; alternative reporters are indicated in red (mCherry) or green (GFP). It should be noted that there was no visible bleedthrough of the mCherry fluorescence into the GFP channel, or vice versa; see also Fig. 4.

**Fig. 2.**
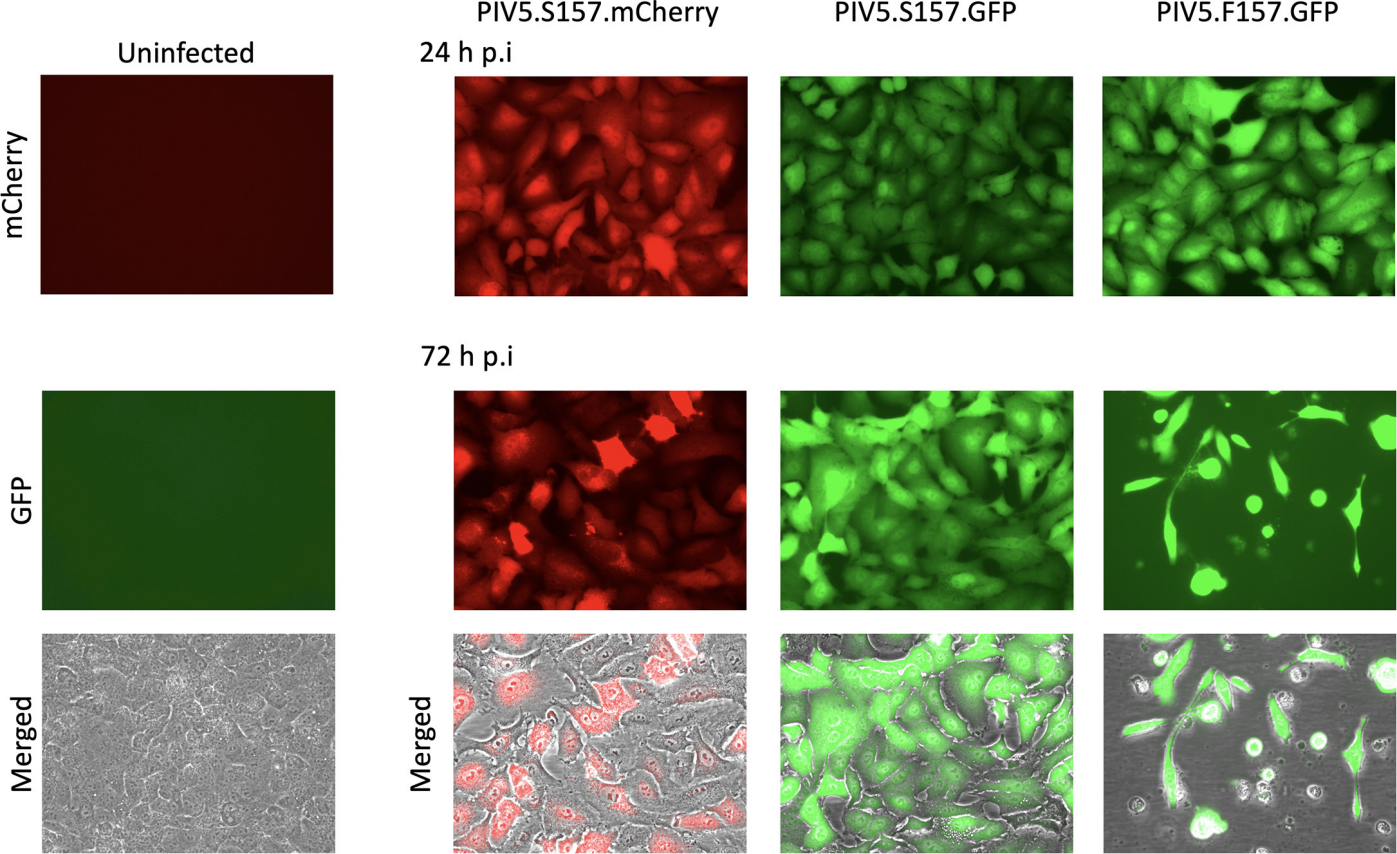
PIV5 reporter viruses expressing either mCherry or GFP with persistent phenotypes (PIV5.S157.mCherry or PIV5.S157.GFP) do not kill A549 cells, whilst the lytic reporter virus expressing GFP (PIV5.F157.GFP) killed the majority of cells by 72 h p.i. Confluent monolayers of A549 cells in 25 cm^2^ flasks (approximately 2×10^6^ cells per flask) were, or were not, infected with 5 p.f.u./cell of PIV5.S157.mCherry,PIV5.S157.GFP or PIV5.F157.GFP for 1 h with rocking, washed three times with prewarmed medium and reincubated at 37 °C with 4 ml of culture medium contain 2 % foetal bovine serum (FBS). Phase contrast, mCherry and GFP fluorescence images of infected and uninfected monolayers were taken at 24 and 72 h p.i. Individual mCherry and GFP images are shown, together with their images merged with a corresponding phase contrast image.

We have previously shown that substitution of a serine (S) at position 157 on the P protein to a phenylalanine (F) changes the W3A strain of PIV5 from a persistent to a lytic phenotype [39]. To further our studies on the relationship between persistent and lytic viruses, we constructed and rescued persistent (PIV5.S157.mCherry and PIV5.S157.GFP) and lytic (PIV5.F157.GFP) reporter viruses that express either mCherry or GFP as described in the Methods section (Fig. 1). To characterize their interaction with cultured cells, A549 cells were infected at a high multiplicity with the individual reporter viruses, and the expression and survival of the cells was visualized at various time post-infection (p.i.). As can be seen in Fig. 2, at 24 h p.i. all infected cells were alive and expressed either mCherry or GFP. The level of mCherry or GFP expression in individual cells within an infected population was similar, although the level of GFP expression in cells infected with PIV5.F157.GFP was slightly higher than in cells infected with PIV5.S157.GFP. However, by 72 h p.i. the majority of cells infected with PIV5.F157.GFP (lytic phenotype) were either dead or dying, whilst those infected either with PIV5.S157.mCherry or PIV5.S157.GFP (persistent phenotypes) were alive and positive for mCherry or GFP, respectively. Unlike what was seen at 24 h p.i. the levels of mCherry and GFP fluorescence at 72 h in cells infected with PIV5.S157.mCherry or PIV5.S157.GFP (persistent phenotypes) varied between cells. Such variation would be predicted, as we have previously shown that virus transcription and replication is switched off as virus persistence is established, and thereafter in persistently infected cells the virus fluxes between active and repressed states in individual cells [39]. Of the few surviving cells infected with PIV5.F157.GFP (lytic phenotype), at 72 h p.i. the level of GFP expression remained high.

### The persistent phenotype is dominant

**Fig. 3.**
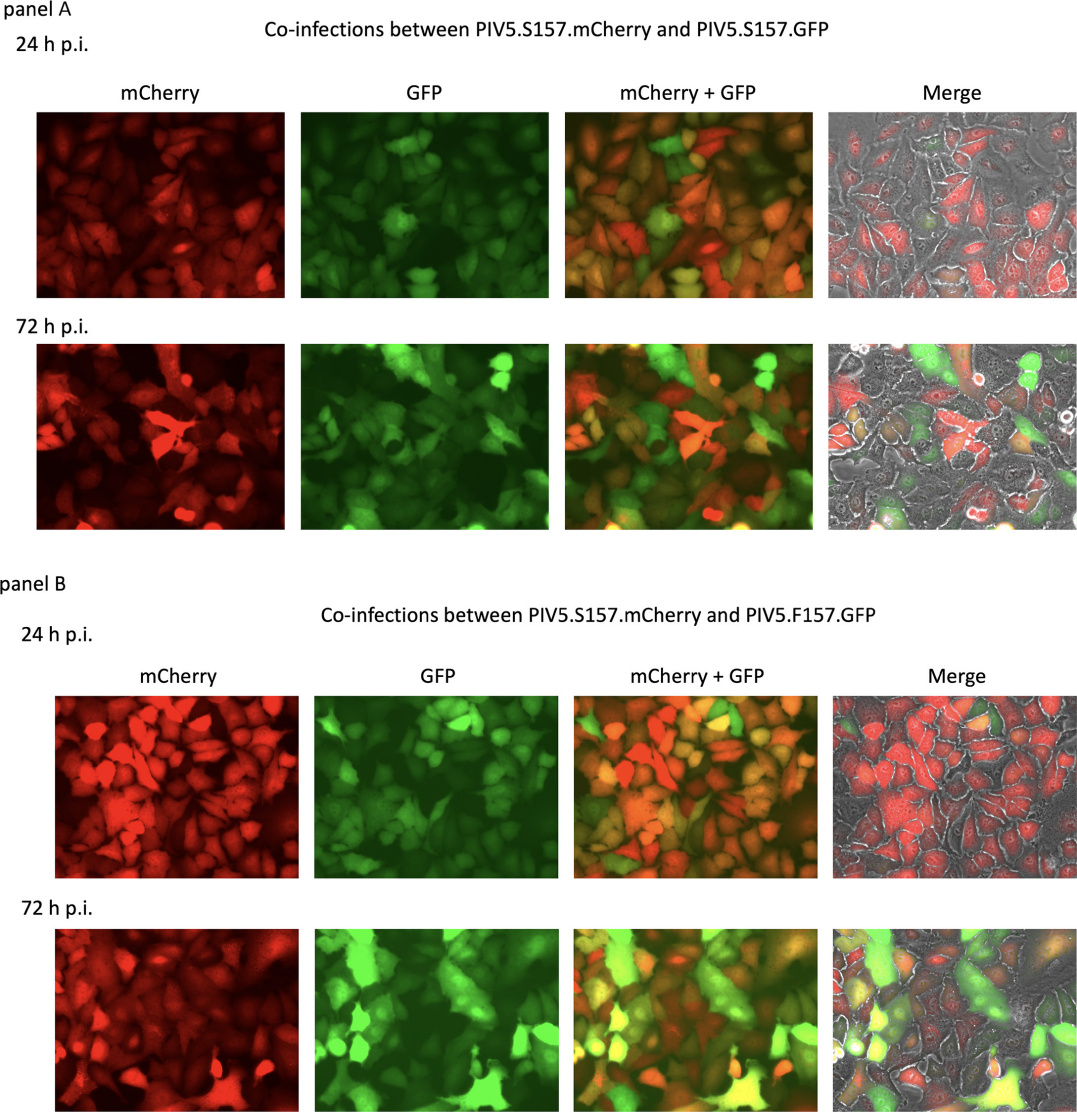
In a parallel set of infections to those described in Fig. 2, monolayers of A549 cells were co-infected with 5 p.f.u./cell of each of the reporter viruses with a persistent phenotype, namely PIV5.S157.mCherry and PIV5.S157.GFP (a), or with persistent and lytic phenotypes, PIV5.S157.mCherry and PIV5.F157.GFP (b). Phase contrast, mCherry and GFP fluorescence images of infected monolayers were taken at 24 and 72 h p.i. Individual mCherry and GFP images are shown, together with combined mCherry and GFP images and the combined images further merged with a corresponding phase contrast image.

**Fig. 4.**
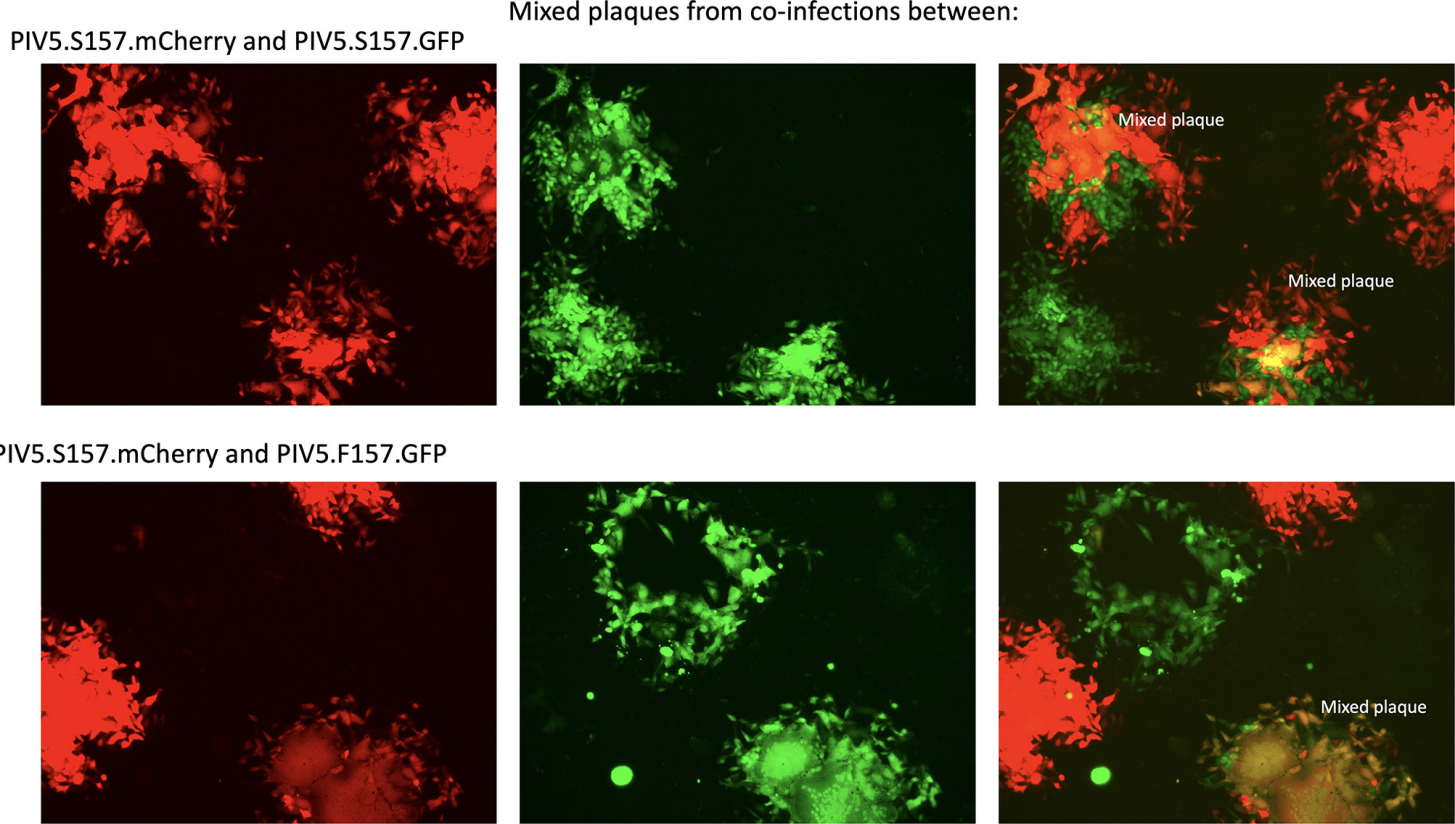
Examples of 3 day plaques derived from virus released from cells co-infected with PIV5.S157.mCherry and PIV5.S157.GFP (persistent phenotypes) or PIV5.S157.mCherry and PIV5.F157.GFP (persistent and lytic phenotypes). Individual mCherry and GFP images are shown, together with their merged images. The virus was present in the medium of cells shown in Fig. 3a, b.

To better understand PIV5 persistence and the biological interactions that may occur between variants with persistent or lytic phenotypes, we next determined whether the lytic or persistent phenotype was dominant in co-infections. In a parallel series of infections to those described above, cells were co-infected at a high multiplicity of infection (m.o.i.) with either a mixture of PIV5.S157.mCherry and PIV5.S157.GFP (persistent phenotypes) or with a mixture of PIV5.S157.mCherry and PIV5.F157.GFP (persistent and lytic phenotypes, respectively). As expected, at 72 h p.i. cells co-infected with the mixture of persistent viruses remained alive, although surprisingly they expressed varying levels of both mCherry and GFP (Fig. 3a. Note: there was no visible bleedthrough of the mCherry fluorescence into the GFP channel, or vice versa; see also Fig. 4. However, and in complete contrast to cells infected with PIV5.F157.GFP alone (lytic phenotype – see Fig. 2), at 72 h p.i. cells co-infected with PIV5.S157.mCherry and PIV5.F157.GFP (persistent and lytic phenotypes, respectively) remained alive and expressed varying levels of mCherry and GFP (Fig. 3b).

With regard to the levels of expression of mCherry and GFP in cells co-infected with either the mixture of persistent reporter viruses (PIV5.S157.mCherry and PIV5.S157.GFP) or the mixture of the persistent and lytic viruses (PIV5.S157.mCherry and PIV5.F157.GFP), the relative levels of mCherry and GFP at 72 h p.i. varied from cell to cell (this was also seen in cells infected with either of the persistent reporter viruses only; Fig. 2). This heterogenous expression is in marked contrast to cells infected solely with PIV5.F157.GFP (lytic phenotype), where all the surviving cells were strongly positive for GFP. It is also of note that in co-infected cells, although all the cells were positive for both GFP and mCherry, the levels of mCherry and GFP relative to each other varied from cell to cell.Thus some cells were strongly positive for mCherry and only weakly positive for GFP, and vice versa, whilst other cells were equally positive for both mCherry and GFP.

### Up to 20 % of virions released from co-infected cells contain both genotypes

**Fig. 5.**
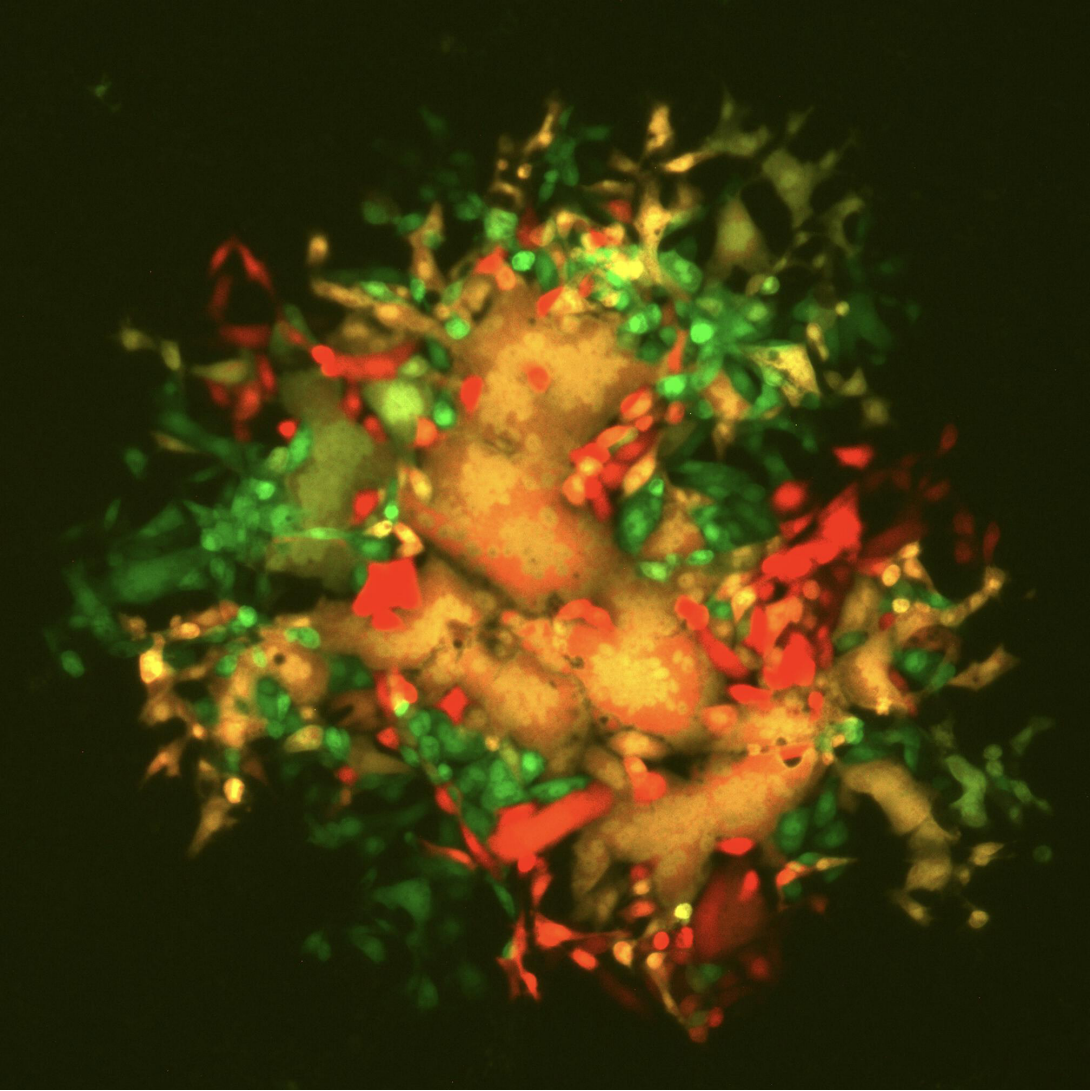
An example of a mixed plaque phenotype of virus derived from passage 2 of cells persistently co-infected with PIV5.S157.mCherry and PIV5.F157.GFP (Fig. 6b). The figure is a combined mCherry and GFP image.

To determine the ratio of the different genotypes released from co-infected cells, the titres of virus released from the cells infected in the above experiments were estimated by plaque assays and were scored as to whether all the cells within the plaque only expressed mCherry or GFP, or whether the plaques had a mixed phenotype, i.e. whether cells within an individual plaque were positive for mCherry, GFP, or both (Table 1). Strikingly, 10–20 % of plaques had a mixed phenotype (Table 1 and Fig. 4). Also strikingly, individual cells within mixed-phenotype plaques were positive for either mCherry or GFP, or both; where cell–cell fusion occurred, the fused cells were generally positive for both mCherry and GFP (Fig. 4, see also Fig. 5). The most likely explanation for these results is that although the initial infected cell within a plaque was infected with a virion that contained both genotypes, as the virus spread virions were released that contained either one or both genotypes, leading to non-fused cells being positive for mCherry, GFP, or both. It is also of note from these experiments that in a co-infection between PIV5.S157.mCherry and PIV5.S157.GFP (persistent phenotypes) and also between PIV5.S157.mCherry and PIV5.F157.GFP (persistent and lytic phenotypes, respectively) approximately equal amounts of both virus genotypes were released (Table 1).

**Table 1.** Titres of infectious virus release at 24 and 72 h p.i. from A549 cells infected at a high m.o.i. with PIV5.S157.mCherry, PIV5.S157.GFP or PIV5.F157.GFP, or co-infected with these viruses as indicated. Plaque assays were used to estimate the amount of infectious virus ml^−1^ culture medium. See legend to Fig. 2 for experimental details. The numbers of plaques positive for mCherry or GFP are shown, and the percentage of those plaques that were positive for both mCherry and GFP is given (% mixed)

24 h p.i.				
	Total	mCherry	GFP	% mixed
S157.mCherry	1×10^7^	1×10^7^		
S157.GFP	1×10^7^		1×10^7^	
F157.GFP	8×10^7^		8×10^7^	
S157.mCherry+ S157.GFP	1.5×10^7^	7×10^6^	8×10^6^	16%
S157.mCherry+ F157.GFP	2.8×10^7^	1×10^7^	1.8×10^7^	12%
**72 h p.i.**				
	Total	mCherry	GFP	% mixed
S157.mCherry	7×10^7^	7×10^7^		
S157.GFP	1.7×10^8^		1.7×10^8^	
F157.GFP	1.8×10^8^		1.8×10^8^	
S157.mCherry+ S157.GFP	1.3×10^8^	6.7×10^7^	6.8×10^7^	11%
S157.mCherry+ F157.GFP	1.8×10^8^	8×10^7^	8.8×10^7^	17%

**Fig. 6.**
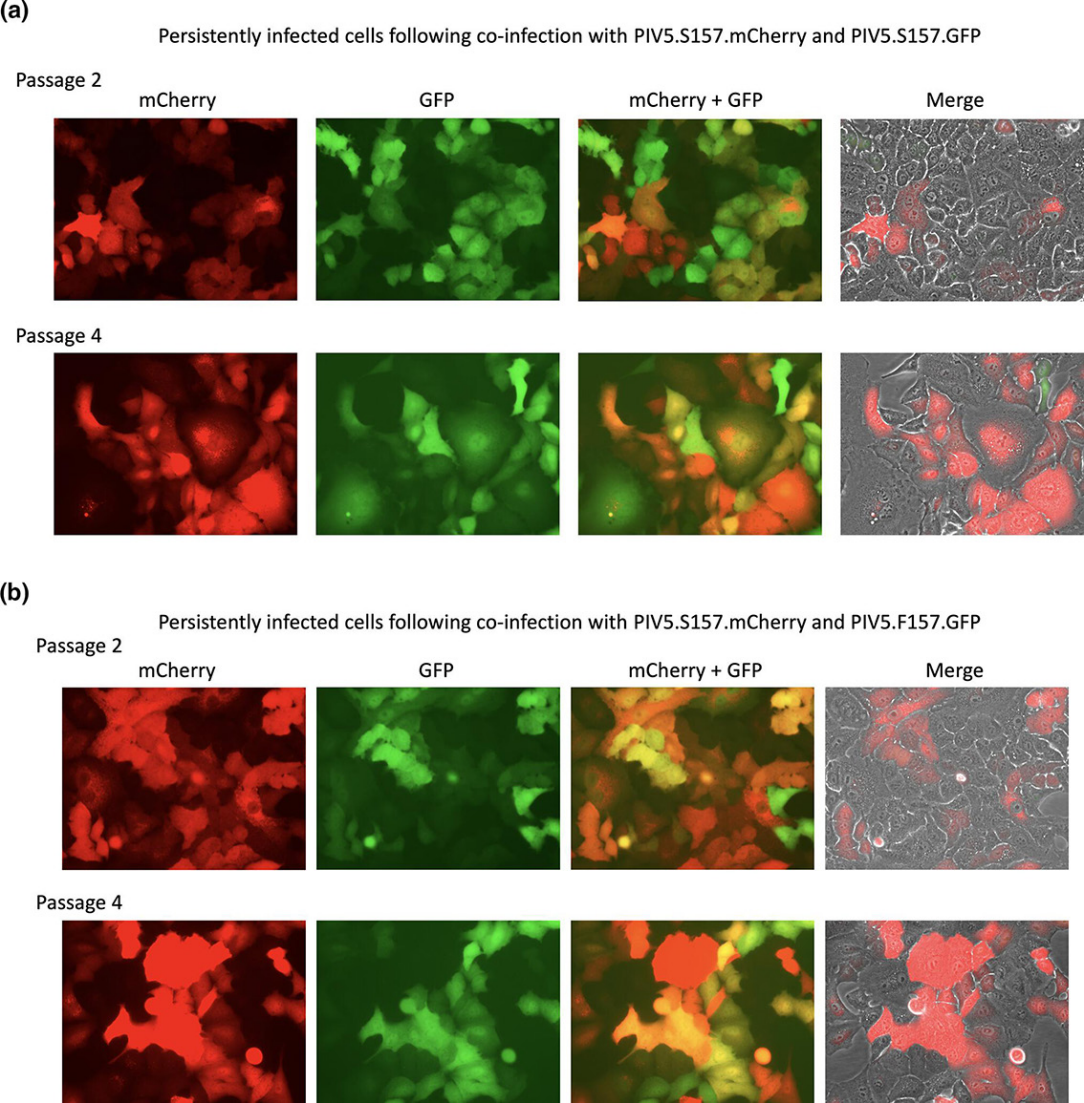
At 96 h p.i. the cells shown in Fig. 3a, b (PIV5.S157.mCherry and PIV5.S157.GFP and PIV5.S157.mCherry and PIV5.F157.GFP, respectively) were passaged and phase contrast, mCherry and GFP fluorescence images of infected monolayers are shown (panels a, b, respectively). Individual mCherry and GFP images are shown, together with combined mCherry and GFP images and the combined images further merged with a corresponding phase contrast image.

### Co-infected cells become persistently infected and continue to harbour both genotypes

We next determined whether persistently infected cells that had been co-infected with both lytic and persistent viruses continue to harbour both genotypes upon passage. At 96 h p.i.persistently infected cells from the above experiment (Fig. 3) were diluted 1 : 4 and passaged. After passages 2 and 4, GFP and mCherry fluorescence was determined. In persistently infected cells, cells that were positive for mCherry and GFP were readily visible, including in cells that had been co-infected with persistent (PIV5.S157.mCherry) and lytic (PIV5.F157.GFP) viruses (Fig. 6a, b). Furthermore, and as would be predicted from our previous results, which showed that in persistently infected cells the virus fluxes between active and repressed states, the level of mCherry and GFP varied from cell to cell. As observed at 72 h p.i. (Fig. 3), the relative levels of expression of mCherry and GFP varied from cell to cell (Fig. 6a, b). In cells co-infected with PIV5.S157.mCherry and PIV5.S157.GFP (both persistent phenotype viruses), it also appeared that a few cells were only positive for either mCherry or GFP (Fig. 6a). In contrast, in cells infected with the persistent and lytic phenotypes, PIV5.S157.mCherry and PIV5.F157.GFP, whilst the intensity of mCherry and GFP fluorescence varied from cell to cell, all the cells that were GFP-positive also expressed some mCherry (Fig. 6b).

To determine whether persistently infected cells continued to release infectious virus upon passage, the titre of virus in the medium of a confluent monolayer of passage 2 of the persistently infected cells was determined by plaque assays (Table 2). The amount of infectious virus released following passage was three to six times lower than the amount of virus released into the medium at 72 h p.i. following initial infection (compare to titres in Table 1). Importantly, these results clearly showed that both infectious lytic (PIV5.F157.GFP) and persistent (PIV5.S157.mCherry) viruses continued to be produced by co-infected cells. Again, up to 16 % of the virions released contained both genotypes (Table 2, Fig. 5).

**Table 2.** Titres of infectious virus from passage 2 of persistently infected cells. Titres of infectious virus released from confluent monolayers of passage 2 A549 cells persistently infected with PIV5.S157.mCherry, PIV5.S157.GFP or cells co-infected with PIV5.S157.mCherry and either PIV5.S157.GFP or PIV5.F157.GFP (cells infected with PIV5.F157.GFP could not be passaged as the few surviving cells did not grow). Plaque assays were used to estimate the amount of infectious virus ml^−1^ culture medium. See legend to Fig. 3 for experimental details. The number of plaques positive for mCherry or GFP are shown, and the percentage of those plaques that were positive for both mCherry and GFP is given (% mixed)

	Total	mCherry	GFP	% mixed
S157.mCherry	2×10^7^	2×10^7^		
S157.GFP	2×10^7^		2×10^7^	
F157.GFP	1.6 x10^7 Non-passaged surviving cells^		1.6 x10^7 Non-passaged surviving cells^	
S157.mCherry+ S157.GFP	3.6×10^7^	1.6×10^7^	2×10^7^	14%
S157.mCherry + F157.GFP	3.2×10^7^	1.4×10^7^	1.8×10^7^	16%

**Fig. 7.**
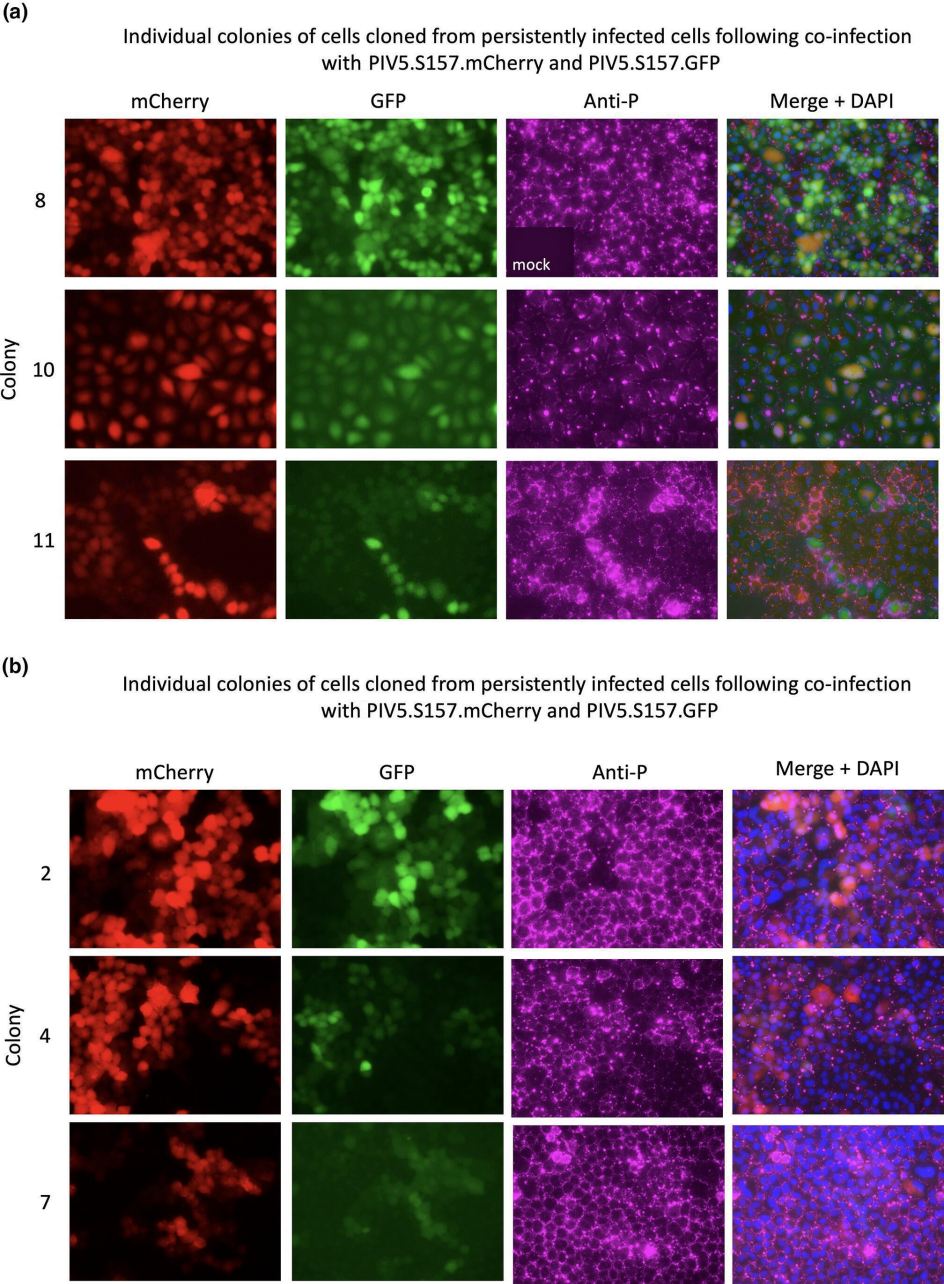
Images of individual colonies of cells cloned from cells persistently infected with PIV5.S157.mCherry and PIV5.S157.GFP (a) or PIV5.S157.mCherry and PIV5.F157.GFP (b). The cells were fixed and indirectly immunostained with anti-P mAb (Cy5-labelled) and cell nuclei visualized by DAPI staining. Individual mCherry, GFP and Cy5 images are shown, together with their merged images and DAPI staining. Note: staining with the anti-P (**v5**) antibody gave no significant background staining of mock-infected cells, as shown in the mock insert in the bottom left-hand panel of the first image of anti-P staining.

To investigate whether persistently co-infected cells continued to harbour both genotypes in individual cells over a prolonged period of passage, the persistently infected cells (above) were cloned in 96-well microtitre plates. Ten colonies were isolated from cells infected with PIV5.S157.mCherry and PIV5.S157.GFP (both persistent), and 10 colonies from cells infected with PIV5.S157.mCherry and PIV5.F157.GFP (persistent and lytic). The cells from the individual colonies were expanded in 25 cm^2^ flasks and the expression of mCherry and GFP was visualized by microscopy. Every colony contained some cells that were clearly positive for GFP and mCherry, although the intensity and number of mCherry/GFP cells in each colony varied, with some colonies containing more cells that were strongly positive for mCherry than strongly positive for GFP, and vice versa. Nevertheless, even in colonies where a high proportion of cells appeared only weakly positive (or even negative) for GFP or mCherry, immunostaining using a monoclonal antibody specific for the P protein showed that all cells remained infected (examples of colonies are shown in Fig. 7a, b). Furthermore, infectious virus was produced by every colony tested, although the yield of each virus varied from colony to colony (Table 3). These data demonstrated that in cells co-infected with both persistent and lytic phenotype viruses, persistence remained the dominant phenotype even after individual cells were cloned and expanded into colonies of cells.

**Table 3.** Titres of infectious virus released from individual clones persistently co-infected cells with either PIV5.S157.mCherry and PIV5.S157.GFP, or PIV5.S157.mCherry and PIV5.F157.GFP. Individual clones were expanded into 25 cm flasks containing 4 ml of medium. When the monolayers were confluent (see Fig. 7 for experimental details) plaque assays were used to estimate the amount of infectious virus ml^−1^ culture medium. The number of plaques positive for mCherry or GFP are shown, and the percentage of those plaques that were positive for both mCherry and GFP is given (% mixed)

Colony	Total	mCherry	GFP	% mixed
S157.mCherry+ S157.GFP				
8	1×10^7^	5×10^6^	5×10^6^	10%
10	4×10^6^	2×10^6^	2×10^6^	7.5%
11	4×10^6^	1×10^6^	3×10^6^	5%
S157.mCherry+ F157.GFP				
4	9×10^6^	4×10^6^	5×10^6^	9%
6	6×10^6^	5×10^6^	1×10^6^	3.3%
7	1.3×10^6^	7×10^5^	6×10^5^	7.6%

## Discussion

We have previously shown that different isolates of PIV5 may have either a lytic or a persistent phenotype and that this phenotype is determined by the phosphorylation status of the RNA polymerase subunit, the P protein. Single-nucleotide substitutions leading to amino acid changes at critical residues in P, for example a serine (which can be phosphorylated) to phenylalanine (which cannot be phosphorylated) change at position 157 in P, results in the switch from a persistent to a lytic phenotype, and vice versa [39]. To further investigate PIV5 persistence we constructed reporter viruses that express either mCherry or GFP that had either lytic or persistent phenotypes. As predicted, reporter viruses with a serine at position 157 (S157) immediately established persistent infections in the absence of co-infection. Additionally, as predicted, the reporter virus with a phenylalanine at position 157 (F157) causes a lytic infection and kills cells. Following infection with persistent reporter viruses, the level of reporter gene expression in individual cells at 24 h p.i. was similar, whereas by 72 h p.i. there was a significant reduction of reporter protein expression in the majority of cells as virus transcription and replication was repressed. In contrast, although by 72 h p.i. the lytic reporter virus (PIV5.F157.GFP) had killed the majority of the cells, the level of GFP expression in surviving cells remained high. These phenotypes are consistent with our previous reports using natural isolates and non-reporter-expressing recombinant viruses [39].

We previously hypothesized that the interplay between persistent and lytic infections plays a major role in virus dynamics and ultimately disease ([41] and discussed in more detail below). Here we show that in co-infections of viruses with persistent and lytic phenotypes the persistent phenotype is dominant. Thus, no obvious cell death was observed in cells co-infected with the lytic and persistent reporter viruses and, strikingly, all cells co-infected with the persistent and lytic reporter viruses continued to harbour both genotypes over a prolonged period of passage. Since the switch between lytic and persistent forms of virus is governed by the phosphorylation status of serine 157 in the P protein, these results strongly suggest that not all the P needs to be phosphorylated in order for downregulation of virus transcription and replication to occur and hence the establishment of persistence. This is consistent with mass spectrometry observations that showed that even in cells infected with a wild-type persistent phenotype virus only a minority of the P protein is phosphorylated at position 157 [39].

Another striking observation of persistently infected cells that had originally been infected with either the two persistent reporter viruses or the persistent and lytic reporter viruses was that the level of GFP and mCherry in the co-infected cells varied from cell to cell, even though the cells were infected at high multiplicity (5–10 p.f.u./cell). Thus, in bulk cultures of persistently infected cells, some cells were more strongly positive for mCherry, others for GFP, and some roughly equally positive for mCherry and GFP. Whilst the explanation for this has not been established, one likely explanation is that upon infection there is asynchrony in the infectious process, leading to some incoming genomes replicating earlier than others. If this is the case, then any incoming virus genome that first establishes productive transcription and replication is likely to dominate the genotype when persistence is established, hence leading to higher expression of that reporter gene. This is supported by the observation that if the bulk population of persistently infected cells is cloned, the ratio of mCherry and GFP in the cloned cells tends not to change significantly, although, as would be expected because virus gene expression fluxes in persistently infected cells, the level of GFP or mCherry fluorescence varies from cell to cell. Another observation from persistently infected cloned cells is that whilst all the cells within a clone are infected, i.e. they can be stained with an antibody to P, the relative level of mCherry and/or GFP varies significantly from clone to clone. It is also of note that PIV5.F157.GFP was detected in, and could be rescued from, all the clones of cells co-infected with the persistent and lytic reporter viruses (PIV5.S157.mCherry and PIV5.F157.GFP: Fig. 7b, Table 3), suggesting that PIV5.S157.mCherry does not rapidly outcompete PIV5.F157.GFP once persistence has been established. We are currently using next-generation sequencing to document virus genome evolution that arises during long-term passage of uncloned and cloned cells persistently infected with PIV5 variants.

A major observation from these studies is that up to ~50 % of PIV5 virions must co-package more than one virus genome. In our experiments, up to 17 % of plaques derived from virus released from co-infected cells had mixed-phenotype plaques (i.e. plaques containing cells that were positive for both mCherry and GFP). Accordingly, the virion that initiated the plaque must have contained both reporter genomes. Therefore, one can reasonably predict that, in addition, a similar proportion of virions must have contained at least two genomes of one or other reporter viruses. This observation, together with the observation that the persistent phenotype is dominant, may have important biological consequences for PIV5 and, by extension, other paramyxoviruses and closely related enveloped viruses. Indeed, the observation that PIV5 virions can contain genomes with different phenotypes also establishes a principle that similar scenarios may occur with other enveloped Mononegavirales. Such phenotypes may not be limited to persistent and lytic, but may, for example, include different genotypes that replicate better in different tissues, as has been proposed to occur in the adaption of measles virus quasispecies to epithelial and lymphocytic cells [44].

We have previously suggested that *in vivo,* depending upon the time post-infection and the development of the adaptive immune response, variants of PIV5 with lytic or persistent phenotypes will be selected rapidly, since only single-amino-acid changes (the result of single-nucleotide substitutions) in P are required to switch a virus from a persistent to a lytic phenotype, and vice versa. Thus, early in infection, lytic phenotypes will be selected because they replicate faster, but as the adaptive immune response develops, viruses with persistent phenotypes will be favoured [39]. This is because any cell that continuously synthesizes high levels of viral proteins will likely be killed either by virus replication or by the adaptive immune response. Therefore, a virus that is able to establish persistence, in which virus gene expression is minimal, may be tolerated by the cell (as is the case *in vitro*) and not be recognized by the immune response [3]. Nevertheless, as shown here and previously, the virus fluxes between active and repressed states in persistently infected cells, and if this occurs *in vivo* persistently infected individuals are likely to release low levels of infectious virus over a prolonged period of time and may act as reservoirs for the virus within a community. In our original model we envisaged that persistently infected individuals would only shed virus with a persistent genotype followed by the rapid selection of lytic genotypes in the newly infected individuals; hence the process of selecting new variants, arising during virus replication, will be required continuously. However, the results presented here suggest that the generation of new variants may not always be required, as persistently infected cells can maintain and release viruses with both a persistent and a lytic phenotype. Furthermore, a high proportion of virions from persistently co-infected cells contain multiple genomes with different phenotypes. The fact that PIV5 virions can contain multiple genomes with different phenotypes may also have other biological consequences. For example, co-infection of a host with a population of viruses with both lytic and persistent variants may reduce the disease severity compared to infection with virus population that primarily has a lytic phenotype, thereby potentially being one of the many factors that influence disease severity in PIV5 (and other paramyxovirus) infections.

## Methods

### Cells and viruses

BSRT7 [45], Vero and A549 cells (from the European Collection of Authenticated Cell Cultures; ECACC) were grown at 37 °C as monolayers in 25 or 75 cm^2^ cell culture flasks, in Dulbecco’s modified Eagle’s medium (DMEM) supplemented with 5 % (BSRT7) or 10 % (v/v) foetal bovine serum (FBS) at 37 °C. Stocks of the PIV5 reporter viruses were grown and titrated in Vero cells.

### Construction and characterization of mCherry and GFP reporter viruses with a lytic or persistent phenotypes

The plasmid pBH276 contains the full-length genomic sequence of the laboratory strain (W3A) of PIV5 inserted between the bacteriophage T7 promoter and a hepatitis delta virus (HDV) ribozyme in a pUC19 vector [46]. The W3A strain of PIV5 has serine at P position 157 (S157). To make PIV5.S157.mCherry, a cassette of the open reading frame of mCherry flanked on the 5′ side by PIV5 nucleotides 1762 to 1814 (containing the 3′ end of the NP gene, the NP–P intergenic sequence and the 5′UTR of the P gene) was constructed by oligonucleotide-directed PCR, incorporating NcoI-compatible ends; this cassette was then inserted into the NcoI site of the PIV5 genome in pBH276 at position 8331. The mCherry gene in PIV5.S157.mCherry is therefore placed between the HN and L genes, and under the control of the transcriptional sequences at the NP–P junction and the 5′UTR of the P gene. PIV5.S157.GFP was constructed from PIV5.S157.mCherry by substituting the mCherry open reading frame with that of GFP. PIV5.F157.GFP was constructed from PIV5.S157.GFP by oligonucleotide-directed mutagenesis of the P gene and replacement of the AgeI–SmaI fragment of PIV5.S157.GFP at W3A with the altered fragment. All plasmids were designed to obey the rule of six. DNA sequences of all PCR-generated material were confirmed by Sanger sequencing (Source Bioscience).

Viruses were rescued by co-transfecting 1 µg of either PIV5.S157.mCherry, PIV5.S157.GFP or GFP. PIV5.F157.GFP with pCAGGS-based helper plasmids directing the synthesis of PIV5-NP (100 ng), PIV5-P (100 ng) and PIV5-L (500 ng) into six-well dishes containing ~10^6^ BSRT7 cells using Lipofectamine – LTX/Plus (Thermo Fisher). Recovery was monitored by tracking the expression of mCherry or GFP and stocks of virus were amplified by two successive passages at low multiplicity of infection (m.o.i.) in Vero cells.

### Visualization of mCherry- and GFP-expressing cells and plaques, and immunofluorescence

Cells expressing mCherry or GFP, including cells in viral plaques that were unfixed and washed [once with complete phosphate-buffered saline (PBS)], were visualized using an EVOS M5000 Cell Imaging System with Plan achromat 4, 10 or 20× objectives and mCherry or GFP Light Cubes. Procedures for immunofluorescence on fixed and permeabilized cells grown on coverslips have been described previously [47, 48]. To visualize the PIV5 P protein, a directly conjugated monoclonal antibody (Alexa Fluor 647 cat. #451 098 InVitrogen) to the V5 tag (which was originally referred to as the Pk tag and was derived from PIV5 [49]) was used.

## References

[1] Lamb RAaGDP, Howley PM (2013). Fields Virology.

[2] Randall RE, Russell WC, Kingsbury DW (1991). The Paramyxoviruses.

[3] Randall RE, Griffin DE (2017). Within host RNA virus persistence: mechanisms and consequences. Curr Opin Virol.

[4] Agrati C, Bartolini B, Bordoni V, Locatelli F, Capobianchi MR (2023). Emerging viral infections in immunocompromised patients: a great challenge to better define the role of immune response. Front Immunol.

[5] Greninger AL, Rybkina K, Lin MJ, Drew-Bear J, Marcink TC (2021). Human parainfluenza virus evolution during lung infection of immunocompromised individuals promotes viral persistence. J Clin Invest.

[6] Muchmore HG, Parkinson AJ, Humphries JE, Scott EN, McIntosh DA (1981). Persistent parainfluenza virus shedding during isolation at the South Pole. Nature.

[7] Buchanan R, Bonthius DJ (2012). Measles virus and associated central nervous system sequelae. Semin Pediatr Neurol.

[8] Sieg M, Heenemann K, Rückner A, Burgener I, Oechtering G (2015). Discovery of new feline paramyxoviruses in domestic cats with chronic kidney disease. Virus Genes.

[9] Sharp CR, Nambulli S, Acciardo AS, Rennick LJ, Drexler JF (2016). Chronic infection of domestic cats with feline morbillivirus, United States. Emerg Infect Dis.

[10] Parks GD, Manuse MJ, Johnson JB, Samal SK (2011). The Biology of Paramyxoviruses.

[11] Robbins SJ, Wrzos H, Kline AL, Tenser RB, Rapp F (1981). Rescue of a cytopathic paramyxovirus from peripheral blood leukocytes in subacute sclerosing panencephalitis. J Infect Dis.

[12] Mitchell DN, Porterfield JS, Micheletti R, Lange LS, Goswami KK (1978). Isolation of an infectious agent from bone-marrows of patients with multiple sclerosis. Lancet.

[13] Goswami KK, Lange LS, Mitchell DN, Cameron KR, Russell WC (1984). Does simian virus 5 infect humans?. J Gen Virol.

[14] Rima BK, Gatherer D, Young DF, Norsted H, Randall RE (2014). Stability of the parainfluenza virus 5 genome revealed by deep sequencing of strains isolated from different hosts and following passage in cell culture. J Virol.

[15] Hsiung GD (1972). Parainfluenza-5 virus. Infection of man and animal. Prog Med Virol.

[16] Choppin PW (1964). Multiplication of a myxovirus (Sv5) with minimal cytopathic effects and without interference. Virology.

[17] Hull RN, Minner JR, Smith JW (1956). New viral agents recovered from tissue cultures of monkey kidney cells. I. Origin and properties of cytopathogenic agents S.V.1, S.V.2, S.V.4, S.V.5, S.V.6, S.V.11, S.V.12 and S.V.15. Am J Hyg.

[18] Ellis JA, Krakowka GS (2012). A review of canine parainfluenza virus infection in dogs. J Am Vet Med Assoc.

[19] Wüthrich D, Boujon CL, Truchet L, Selimovic-Hamza S, Oevermann A (2016). Exploring the virome of cattle with non-suppurative encephalitis of unknown etiology by metagenomics. Virology.

[20] Hierweger MM, Werder S, Seuberlich T (2020). Parainfluenza virus 5 infection in neurological disease and encephalitis of cattle. Int J Mol Sci.

[21] Kim J-M, Kim H-R, Jeon G-T, Baek J-S, Kwon O-D (2023). Molecular detection of porcine parainfluenza viruses 1 and 5 using a newly developed duplex real-time RT-PCR in South Korea. Animals.

[22] Singh F, Rajukumar K, Senthilkumar D, Venkatesh G, Srivastava D (2022). First report on co-isolation and whole-genomic characterisation of mammalian orthorubulavirus 5 and mammalian orthoreovirus type 3 from domestic pigs in India. Arch Virol.

[23] Heinen E, Herbst W, Schmeer N (1998). Isolation of a cytopathogenic virus from a case of porcine reproductive and respiratory syndrome (PRRS) and its characterization as parainfluenza virus type 2. Arch Virol.

[24] Ibrahim YM, Zhang W, Werid GM, Zhang H, Pan Y (2022). Characterization of parainfluenza virus 5 from diarrheic piglet highlights its zoonotic potential. Transbound Emerg Dis.

[25] Truong H-T, Nguyen V-G, Pham L-B-H, Huynh T-M-L, Lee J (2023). PCR-based detection and genetic characterization of parainfluenza virus 5 detected in pigs in Korea from 2016 to 2018. Vet Sci.

[26] Cui X, Fan K, Liang X, Gong W, Chen W (2023). Virus diversity, wildlife-domestic animal circulation and potential zoonotic viruses of small mammals, pangolins and zoo animals. Nat Commun.

[27] Zhai J-Q, Zhai S-L, Lin T, Liu J-K, Wang H-X (2017). First complete genome sequence of parainfluenza virus 5 isolated from lesser panda. Arch Virol.

[28] Wang X, Chen W, Xiang R, Li L, Chen J (2019). Complete genome sequence of Parainfluenza Virus 5 (PIV5) from a sunda pangolin (*Manis javanica*) in China. J Wildl Dis.

[29] Johnson RI, Tachedjian M, Rowe B, Clayton BA, Layton R (2018). Alston virus, a novel paramyxovirus isolated from bats causes upper respiratory tract infection in experimentally challenged ferrets. Viruses.

[30] Chen Z, Xu P, Salyards GW, Harvey SB, Rada B (2012). Evaluating a parainfluenza virus 5-based vaccine in a host with pre-existing immunity against parainfluenza virus 5. PLoS One.

[31] Cohn ML, Robinson ED, Thomas D, Faerber M, Carey S (1996). T cell responses to the paramyxovirus simian virus 5: studies in multiple sclerosis and normal populations. Pathobiology.

[32] Lee YN, Park C-K, Kim S-H, Lee DS, Shin J-H (2013). Characterization in vitro and in vivo of a novel porcine parainfluenza virus 5 isolate in Korea. Virus Res.

[33] Jiang N, Wang E, Guo D, Wang X, Su M (2018). Isolation and molecular characterization of parainfluenza virus 5 in diarrhea-affected piglets in China. J Vet Med Sci.

[34] Liu Y, Li N, Zhang S, Zhang F, Lian H (2015). Parainfluenza virus 5 as possible cause of severe respiratory disease in calves, China. Emerg Infect Dis.

[35] Young DF, Carlos TS, Hagmaier K, Fan L, Randall RE (2007). AGS and other tissue culture cells can unknowingly be persistently infected with PIV5; a virus that blocks interferon signalling by degrading STAT1. Virology.

[36] Feehan BJ, Penin AA, Mukhin AN, Kumar D, Moskvina AS (2019). Novel *Mammalian orthorubulavirus 5* discovered as accidental cell culture contaminant. Viruses.

[37] Goswami KK, Cameron KR, Russell WC, Lange LS, Mitchell DN (1984). Evidence for the persistence of paramyxoviruses in human bone marrows. J Gen Virol.

[38] Wignall-Fleming EB, Hughes DJ, Vattipally S, Modha S, Goodbourn S (2019). Analysis of paramyxovirus transcription and replication by high-throughput sequencing. J Virol.

[39] Young DF, Wignall-Fleming EB, Busse DC, Pickin MJ, Hankinson J (2019). The switch between acute and persistent paramyxovirus infection caused by single amino acid substitutions in the RNA polymerase P subunit. PLoS Pathog.

[40] Cox RM, Plemper RK (2017). Structure and organization of paramyxovirus particles. Curr Opin Virol.

[41] Loney C, Mottet-Osman G, Roux L, Bhella D (2009). Paramyxovirus ultrastructure and genome packaging: cryo-electron tomography of sendai virus. J Virol.

[42] Rager M, Vongpunsawad S, Duprex WP, Cattaneo R (2002). Polyploid measles virus with hexameric genome length. EMBO J.

[43] Goff PH, Gao Q, Palese P (2012). A majority of infectious Newcastle disease virus particles contain a single genome, while a minority contain multiple genomes. J Virol.

[44] Donohue RC, Pfaller CK, Cattaneo R (2019). Cyclical adaptation of measles virus quasispecies to epithelial and lymphocytic cells: To V, or not to V. PLoS Pathog.

[45] Buchholz UJ, Finke S, Conzelmann KK (1999). Generation of bovine respiratory syncytial virus (BRSV) from cDNA: BRSV NS2 is not essential for virus replication in tissue culture, and the human RSV leader region acts as a functional BRSV genome promoter. J Virol.

[46] He B, Paterson RG, Ward CD, Lamb RA (1997). Recovery of infectious SV5 from cloned DNA and expression of a foreign gene. Virology.

[47] Randall RE, Dinwoodie N (1986). Intranuclear localization of herpes simplex virus immediate-early and delayed-early proteins: evidence that ICP 4 is associated with progeny virus DNA. J Gen Virol.

[48] Carlos TS, Fearns R, Randall RE (2005). Interferon-induced alterations in the pattern of parainfluenza virus 5 transcription and protein synthesis and the induction of virus inclusion bodies. J Virol.

[49] Hanke T, Szawlowski P, Randall RE (1992). Construction of solid matrix-antibody-antigen complexes containing simian immunodeficiency virus p27 using tag-specific monoclonal antibody and tag-linked antigen. J Gen Virol.

